# Intra-pulse difference frequency generation in ZnGeP_2_ for high-frequency terahertz radiation generation

**DOI:** 10.1038/s41598-023-35131-6

**Published:** 2023-05-19

**Authors:** B. N. Carnio, M. Zhang, K. T. Zawilski, P. G. Schunemann, O. Moutanabbir, A. Y. Elezzabi

**Affiliations:** 1 Department of Engineering Physics, École Polytechnique de Montréal, Succ. Centre-Ville, C. P. 6079, Montreal, QC H3C 3A7 Canada; 2 Department of Electrical Engineering, University of Alberta, Edmonton, AB T6G 2V4 Canada; 3 BAE Systems, MER15-1813, P.O. Box 868, Nashua, NH 03061 USA

**Keywords:** Nonlinear optics, Optics and photonics, Optical materials and structures

## Abstract

The highly-nonlinear chalcopyrite crystal family has experienced remarkable success as source crystals in the mid-infrared spectral range, such that these crystals are primary candidates for producing high terahertz frequency (i.e., $\gtrsim$ 10 THz) electric fields. A phase-resolved terahertz electric field pulse is produced via intra-pulse difference frequency generation in a chalcopyrite (110) ZnGeP_2_ crystal, with phase-matching being satisfied by the excitation electric field pulse having polarizations along both the ordinary and extraordinary crystal axes. While maximum spectral power is observed at the frequency of 24.5 THz (in agreement with intra-pulse phase-matching calculations), generation nonetheless occurs across the wide spectral range of 23–30 THz. To our knowledge, this is the first time a chalcopyrite ZnGeP_2_ crystal has been used for the generation of phase-resolved high-frequency terahertz electric fields.

## Introduction

For decades, terahertz (THz) technologies have been gaining interest, in part from their ability to realize electric field waveforms in the time-domain (i.e. phase-resolved or field-resolved capabilities). Such a property grants THz technologies with a key advantage, critical to both academic research and practical applications^1–3^. With the continuing advancement of THz technologies, THz radiation generation and detection techniques have realized spectral components extending into the mid-infrared regime^4^. Given this spectral overlap, it is informative to compare attributes between the THz and mid-infrared spectral ranges. The THz frequency regime is dominated by phase-resolved emission and sensing techniques, whereas the mid-infrared frequency regime is dominated by techniques only providing magnitude (i.e., intensity) information. Additionally, a single broadband excitation laser pulse is typically used to produce broadband THz electric fields, whereas two separate narrowband excitation electric fields are often used to produce narrowband mid-infrared radiation. Consequently, the THz radiation phase-matching conditions are more restrictive, as phase-matching must be satisfied by the spectral components present within the excitation pulse (i.e., intra-pulse phase-matching). Despite these differences, emission and sensing crystals used in the mid-infrared regime could be adapted for use in the high-frequency THz regime (i.e. defined as frequencies up to, and even exceeding, ~ 40 THz^5–8^), due to the fact that the upper limit of the THz regime overlaps with the lower-limit of the mid-infrared regime. Given that highly-nonlinear chalcopyrite crystals (e.g., ZnGeP_2_, CdSiP_2_, AgGaSe_2_, AgGaS_2_, etc.) have found great success in the mid-infrared regime^9–12^ (not to mention the low THz frequency regime^13^), these crystals are good candidates for the emission and sensing of phase-resolved electric fields in the high-frequency THz spectral range. Such chalcopyrite crystals are structurally robust, in contrast to the soft and flaky nature of GaSe (i.e. a commonly-utilized crystal used for generation and detection of phase-resolved electric fields between ~ 10 and 40 THz^5–8^). Crystals exhibiting robust physical and mechanical properties are needed for practical applications, particularly those applications devised for use in uncontrolled or harsh environments. Therefore, advancements in high-frequency THz technologies for practical applications rely on the discovery of emission and sensing nonlinear crystals novel to this spectral regime. When investigating such crystals in the THz frequency regime, special attention must be given to phase-resolved emission and detection capabilities, as well as determining intra-pulse phase-matching conditions.

Herein, a (110) ZnGeP_2_ chalcopyrite crystal is used for the generation of a phase-resolved THz electric field pulse, encompassing frequency components between 23 and 30 THz. Intra-pulse phase-matching is achieved using an excitation electric field pulse having polarization components along both the ordinary and extraordinary axes of the ZnGeP_2_ crystal. In agreement with intra-pulse phase-matching calculations, maximum generation is observed at the frequency of 24.5 THz. To our knowledge, this is the first investigation in which a chalcopyrite ZnGeP_2_ crystal (prominent for generation within the mid-infrared regime) has been used to realize phase-resolved radiation in the high-frequency THz spectral regime. As such, this work is envisioned to stimulate further investigations, primarily in the use of other chalcopyrite crystals for high-frequency phase-resolved THz radiation approaches.

## Crystal characteristics and nonlinear properties

**Figure 1:**
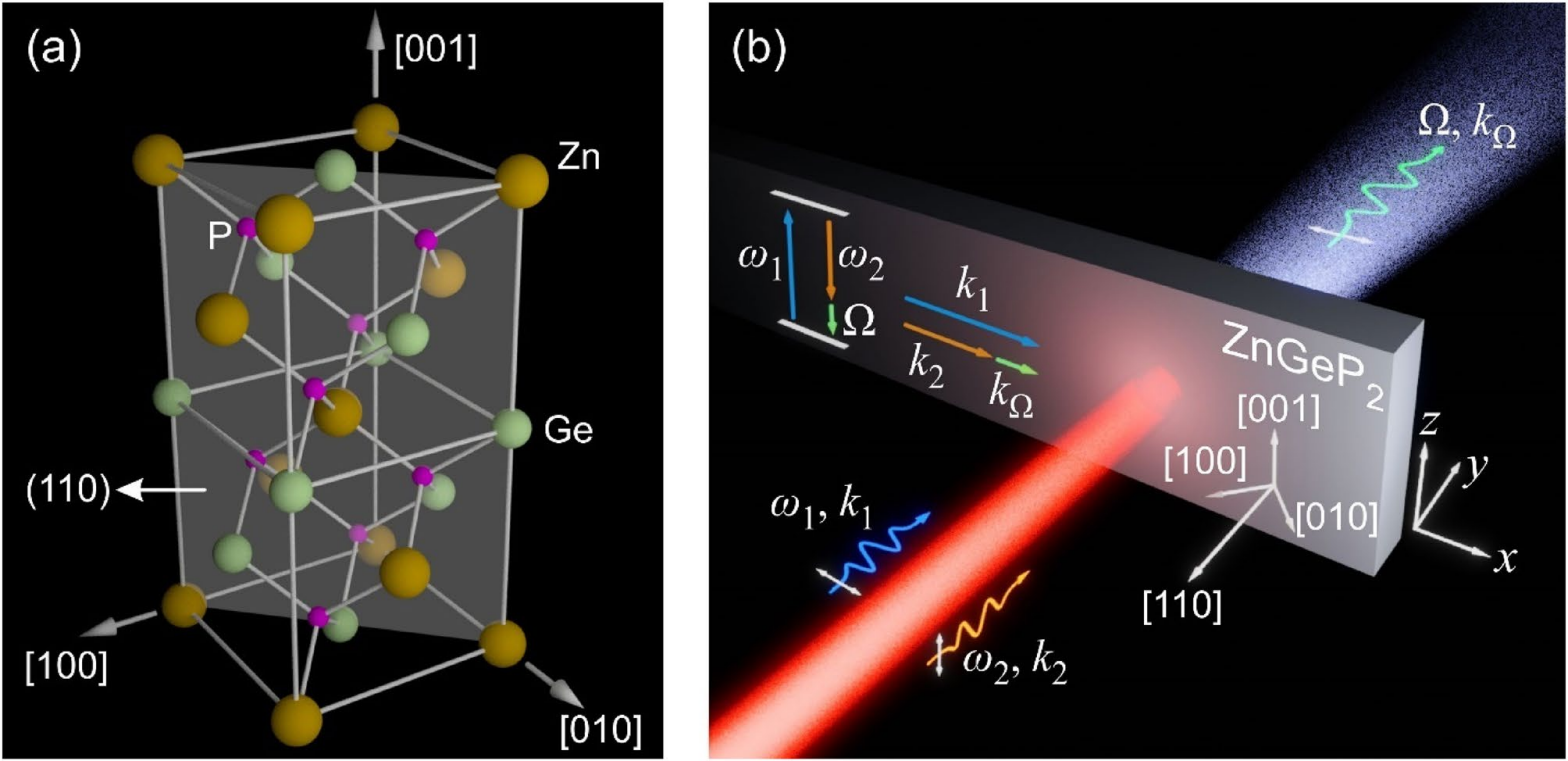
(**a**) Illustration of the ZnGeP_2_ unit cell and (110) cut plane. (**b**) Photon-picture illustration of the DFG process in a (110) ZnGeP_2_ crystal. The energy conservation condition (i.e. ${\omega }_{1}={\omega }_{2}+\Omega$) is illustrated, along with the perfect phase-matching momentum conservation condition (i.e. ${k}_{1}={k}_{2}+{k}_{\Omega }$) described by Eq. (7).

Of all the chalcopyrite crystals used for generation in the mid-infrared spectral regime, ZnGeP_2_ has received the majority of interest^9,14–17^. The ZnGeP_2_ chalcopyrite unit cell structure is depicted in Fig. 1a, which contains four Zn atoms, four Ge atoms, and eight P atoms^18^. The ZnGeP_2_ unit cell lattice exhibits a tetragonal crystal system, being defined by the dimensions of 10.766 Å along the [001] axis and 5.465 Å along the [100] and [010] axes^19^. As seen from Fig. 1a, ZnGeP_2_ does not exhibit inversion symmetry (i.e. it is non-centrosymmetric), such that this crystal supports second-order nonlinear effects. Given that ZnGeP_2_ possesses a high mid-infrared second-order nonlinearity^20^, this crystal exhibits great promise as a source for high THz frequency difference frequency generation (DFG) electric fields. DFG is a second-order nonlinear process in which nonlinear dipole oscillations are induced in a nonlinear crystal. Subsequently, coherent electromagnetic radiation is emitted due to the acceleration and deceleration of these charged dipoles^21^. Since ZnGeP_2_ has a $\overline{4 }2m$ point group symmetry^22^, its second-order nonlinear polarization components are represented as^23^:

1 $$\left[\begin{array}{l}{P}_{\left[100\right]}^{(2)}\\ {P}_{\left[010\right]}^{(2)}\\ {P}_{\left[001\right]}^{(2)}\end{array}\right]=2{\varepsilon }_{0}\left[\begin{array}{ll}\begin{array}{ccc}0& 0& 0\\ 0& 0& 0\\ 0& 0& 0\end{array}& \begin{array}{ccc}{d}_{14}& 0& 0\\ 0& {d}_{14}& 0\\ 0& 0& {d}_{36}\end{array}\end{array}\right]\left[\begin{array}{l}\begin{array}{l}{E}_{\left[100\right]}^{2}\\ {E}_{\left[010\right]}^{2}\\ {E}_{\left[001\right]}^{2}\end{array}\\ \begin{array}{l}2{E}_{\left[010\right]}{E}_{\left[001\right]}\\ 2{E}_{\left[100\right]}{E}_{\left[001\right]}\\ 2{E}_{\left[100\right]}{E}_{\left[010\right]}\end{array}\end{array}\right] ,$$

where ${P}_{\left[100\right]}^{(2)}$, ${P}_{\left[010\right]}^{(2)}$, and ${P}_{\left[001\right]}^{(2)}$ represent the second-order nonlinear polarizations induced along the [100], [010], and [001] crystallographic axes of the crystal, respectively, ${E}_{\left[100\right]}$, ${E}_{\left[010\right]}$, and ${E}_{\left[001\right]}$ represent the excitation electric fields having polarizations along the [100], [010], and [001] crystallographic axes of the crystal, respectively, and ${d}_{14}$ and ${d}_{36}$ represent non-vanishing tensor elements. To achieve phase-matching in the mid-infrared regime, a ZnGeP_2_ crystal can be cut along various crystallographic planes^9,14–17^. Herein, a (110) ZnGeP_2_ crystal is considered (see plane depicted in Fig. 1a), as it provides a simple cut orientation having the potential to permit phase-matching in the intra-pulse DFG process (i.e. DFG occurring between the spectral components encompassed within a single excitation pulse). Utilizing a coordinate rotation transformation, Eq. (1) is instead expressed with respect to the cartesian coordinate system indicated in Fig. 1b, giving:

2 $$\left[\begin{array}{l}{P}_{x}^{(2)}\\ {P}_{y}^{(2)}\\ {P}_{z}^{(2)}\end{array}\right]=2{\varepsilon }_{0}\left[\begin{array}{ll}\begin{array}{lll}0& 0& 0\\ 0& 0& 0\\ -{d}_{36}& {d}_{36}& 0\end{array}& \begin{array}{lll}0& -{d}_{14}& 0\\ {d}_{14}& 0& 0\\ 0& 0& 0\end{array}\end{array}\right]\left[\begin{array}{l}\begin{array}{l}{E}_{x}^{2}\\ {E}_{y}^{2}\\ {E}_{z}^{2}\end{array}\\ \begin{array}{l}2{E}_{y}{E}_{z}\\ 2{E}_{x}{E}_{z}\\ 2{E}_{x}{E}_{y}\end{array}\end{array}\right] ,$$

where ${P}_{x}^{(2)}$, ${P}_{y}^{(2)}$, and ${P}_{z}^{(2)}$ represent the second-order nonlinear polarizations induced with respect to the *x*, *y*, and *z* axes, respectively, and ${E}_{x}$, ${E}_{y}$, and ${E}_{z}$ represent the excitation electric fields having polarizations oriented along the *x*, *y*, and *z* axes, respectively. Due to the transverse-electromagnetic nature of electromagnetic waves in a bulk media, ${E}_{y}$ = 0 when exciting the bulk (110) ZnGeP_2_ crystal at normal incidence, such that Eq. (2) simplifies to the following three equations:

3 $${P}_{x}^{(2)}=-4{\varepsilon }_{0}{d}_{14}{E}_{x}{E}_{z} ,$$

4 $${P}_{y}^{(2)}=0 ,$$

5 $${P}_{z}^{(2)}=-2{\varepsilon }_{0}{d}_{36}{E}_{x}^{2} .$$

To satisfy the DFG phase-matching condition, the wavevector associated with the second-order nonlinear polarization must equal the superposition of the excitation electric field wavevectors^24^. Given that Eq. (3) is influenced by separate excitation electric field (i.e. ${E}_{x}$ and ${E}_{z}$) wavevectors and Eq. (5) depends only on a single excitation electric field (i.e. ${E}_{x}$) wavevector, Eq. (3) provides more flexibility in satisfying this phase-matching condition opposed to Eq. (5). As such, Eq. (3) is re-casted to indicate the angular frequencies associated with the excitation electric fields, the induced second-order nonlinear polarization, and the nonlinear tensor element, giving:

6 $${P}_{x}^{(2)}\left(\Omega \right)=-4{\varepsilon }_{0}{d}_{14}\left(\Omega :{\omega }_{1},{\omega }_{2}\right){E}_{x}\left({\omega }_{1}\right){E}_{z}\left({\omega }_{2}\right) ,$$

where ${\omega }_{1}$ is the angular frequency associated with ${E}_{x}$, ${\omega }_{2}$ is the angular frequency associated with ${E}_{z}$, $\Omega$ is the angular frequency associated with ${P}_{x}^{(2)}$, and ${d}_{14}\left(\Omega :{\omega }_{1},{\omega }_{2}\right)$ signifies that ${d}_{14}$ depends on the generated angular frequency (i.e. $\Omega$) and the excitation angular frequencies (i.e. ${\omega }_{1}$ and ${\omega }_{2}$). Figure 1b illustrates the photon-picture of the arrangement dictated by Eq. (6), where a photon having the angular frequency of ${\omega }_{1}$ is polarized along the *x*-axis (experiencing the ordinary refractive index of the crystal) and a photon having the angular frequency of ${\omega }_{2}$ is polarized along the *z*-axis (experiencing the extraordinary refractive index of the crystal). Nonlinear DFG (see Eq. 6) leads to the generation of a photon having the angular frequency of $\Omega ={\omega }_{1}-{\omega }_{2}$, polarized along the *x*-axis and experiencing the crystal’s ordinary refractive index. Here, the angular frequency of ${\omega }_{1}$ is assumed to be higher than the angular frequency of ${\omega }_{2}$, as depicted by the energy conservation diagram in Fig. 1b. The momentum conservation diagram in Fig. 1b illustrates the optimal situation of perfect phase-matching when:

7 $${k}_{1}={k}_{2}+{k}_{\Omega } ,$$

where ${k}_{1}$ [= ${\omega }_{1}{n}_{o}\left({\omega }_{1}\right)/c$] is the wavevector of the photon having the angular frequency of ${\omega }_{1}$, ${k}_{2}$ [= ${\omega }_{2}{n}_{e}\left({\omega }_{2}\right)/c$] is the wavevector of the photon having the angular frequency of ${\omega }_{2}$, ${k}_{\Omega }$ [= $\Omega {n}_{o}\left(\Omega \right)/c$] is the wavevector of the photon having the angular frequency ${\omega }_{\Omega }$, ${n}_{o}$ is the ordinary refractive index of the ZnGeP_2_ crystal, ${n}_{e}$ is the extraordinary refractive index of the ZnGeP_2_ crystal, and* c* is the speed of light. To quantify the degree of phase-matching, or phase-mismatching, the coherence length, ${L}_{c}$, is used:

8 $${L}_{c}=\frac{\pi }{\left|{k}_{1}-{k}_{2}-{k}_{\Omega }\right|} .$$

As expected, ${L}_{c}\to \infty$ for perfect phase-matching [i.e., when Eq. (7) is satisfied].

## Theoretical methodologies

### Coherence length calculations

**Figure 2:**
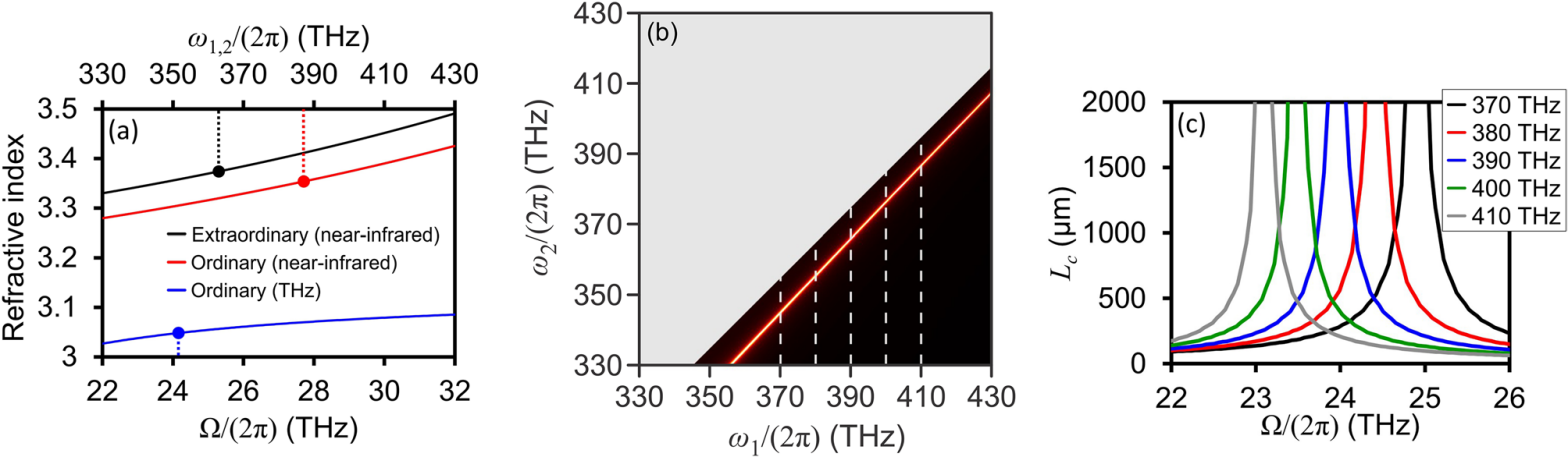
(**a**) Near-infrared ordinary and extraordinary refractive indices, as well as the THz ordinary refractive index, for a ZnGeP_2_ crystal. The closed-circles indicate a representative set of three frequencies that satisfy the perfect phase-matching condition [i.e., Eq. (7)]. (**b**) Intra-pulse DFG coherence length, with perfect phase-matching indicated by the red line. (**c**) Intra-pulse DFG coherence length for fixed ${\omega }_{1}/\left(2\pi \right)$ between 370 and 410 THz [indicated by the dotted lines in (**b**)].

To apply the phase-matching criteria [i.e., Eq. (8)] to intra-pulse DFG in a (110) ZnGeP_2_ crystal, it is necessary to have the ordinary and extraordinary refractive indices at the excitation frequencies, as well as the ordinary refractive index at the generated frequencies. Intra-pulse mixing between the excitation frequency components results in DFG spectral components being produced in the THz frequency regime. Figure 2a depicts the ZnGeP_2_ refractive indices (obtained from Ref. ^25^) in the near-infrared and THz frequency ranges. The closed circles indicate a representative set of three frequencies that satisfy the perfect phase-matching condition (i.e., ${L}_{c}\to \infty$). Here, electric fields at ${\omega }_{1}/\left(2\pi \right)$=387.1 THz and ${\omega }_{2}/\left(2\pi \right)$=363 THz mix to produce phase-matched electric field generation at $\Omega /\left(2\pi \right)$=24.1 THz. Importantly, many other ${\omega }_{1}$, ${\omega }_{2}$, and $\Omega$ frequency combinations also satisfy ${L}_{c}\to \infty$, as depicted by the spectrum in Fig. 2b (with the red line indicating ${L}_{c}\to \infty$). ${L}_{c}$ is calculated at distinct frequencies of ${\omega }_{1}/\left(2\pi \right)$, as specified by the dotted lines in Fig. 2b, and plotted with respect to the frequency of $\Omega /\left(2\pi \right)$ (see Fig. 2c). As ${\omega }_{1}/\left(2\pi \right)$ increases from 370 to 410 THz, the $\Omega /\left(2\pi \right)$ exhibiting ${L}_{c}\to \infty$ decreases from 25 to 23.2 THz. It should be noted that perfect phase-matching is not observed when the higher angular frequency (i.e., ${\omega }_{1}$) electric field component experiences ${n}_{e}$ and the lower angular frequency (i.e., ${\omega }_{2}$) electric field component experiences ${n}_{o}$.

### Nonlinearity dispersion

**Figure 3:**
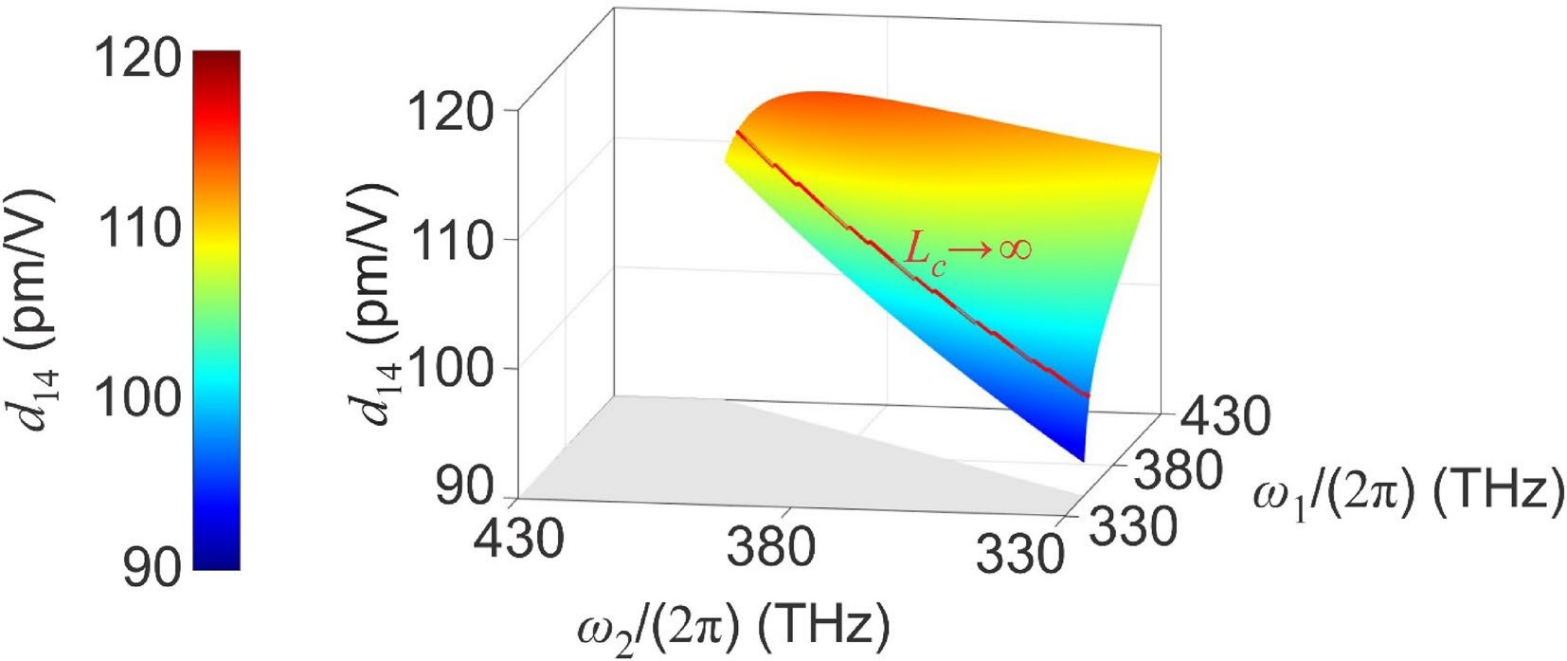
${d}_{14}\left(\Omega :{\omega }_{1},{\omega }_{2}\right)$ obtained using Miller’s rule^26^, along with the experimental data from Refs.^25,27,28^. Recall, energy conservation dictating that $\Omega /\left(2\pi \right)={\omega }_{1}/\left(2\pi \right)-{\omega }_{2}/\left(2\pi \right)$.

In the arrangement illustrated in Fig. 1b, frequency-conversion is influenced by the ${d}_{14}\left(\Omega :{\omega }_{1},{\omega }_{2}\right)$ tensor element via Eq. (6). Given the nonlinear tensor element at a specified frequency set [i.e. ${d}_{14}\left({\omega }_{A}:{\omega }_{B},{\omega }_{C}\right)$, where ${\omega }_{A}$ represents the generated angular frequency and ${\omega }_{B,C}$ represent the excitation angular frequencies], the nonlinear tensor element can be obtained at all other frequencies via Miller’s rule, stated as^26^:

9 $${d}_{14}\left(\Omega :{\omega }_{1},{\omega }_{2}\right)=\frac{{\chi }_{o}\left(\Omega \right){\chi }_{o}\left({\omega }_{1}\right){\chi }_{e}\left({\omega }_{2}\right)}{{\chi }_{o}\left({\omega }_{A}\right){\chi }_{o}\left({\omega }_{B}\right){\chi }_{e}\left({\omega }_{C}\right)}{d}_{14}\left({\omega }_{A}:{\omega }_{B},{\omega }_{C}\right) ,$$

where ${\chi }_{o,e}$[= ${n}_{o,e}^{2}-1$] is the ordinary and extraordinary linear susceptibilities, respectively. ${d}_{14}\left({\omega }_{A}:{\omega }_{B},{\omega }_{C}\right)$=75 pm/V at ${\omega }_{A}/\left(2\pi \right)$=62.5 THz, ${\omega }_{B}/\left(2\pi \right)$=31.3 THz, and ${\omega }_{C}/\left(2\pi \right)$=31.3 THz^27,28^. Using this ${d}_{14}\left({\omega }_{A}:{\omega }_{B},{\omega }_{C}\right)$, along with the linear optical data in Ref. ^25^, ${d}_{14}\left(\Omega :{\omega }_{1},{\omega }_{2}\right)$ is obtained via Eq. (9) and shown in Fig. 3. We are particularly interested in the ZnGeP_2_ nonlinearity at frequencies exhibiting ${L}_{c}\to \infty$, since these are the primary spectral components contributing to the intra-pulse DFG process. The solid red line in Fig. 3 indicates frequencies at which ${L}_{c}\to \infty$ [as determined from Fig. 2b], such that ${d}_{14}\left(\Omega :{\omega }_{1},{\omega }_{2}\right)$ is high, monotonically ranging between 97 and 111 pm/V. Worth noting is that a (012) ZnGeP_2_ crystal (known to have a higher nonlinearity than ZnGeP_2_ crystals cut along other planes^29^) was also considered. However, perfect phase-matching was not observed for ${\omega }_{1}/\left(2\pi \right)$ and ${\omega }_{2}/\left(2\pi \right)$ between 330 and 430 THz, whereas the (110) ZnGeP_2_ crystal both: (i) satisfies the perfect phase-matching criterion and (ii) provides a nonlinearity of nearly 100 pm/V.

## Experimental method, results, and discussion

### THz time-domain spectroscopy

To experimentally-realize the arrangement depicted in Fig. 1b, a 990 µm-thick, (110) ZnGeP_2_ crystal is excited using an 800 nm-central wavelength pulse produced from a Ti:sapphire laser oscillator. Using a half waveplate, the excitation pulse polarization is rotated to obtain a linear polarization oriented at an angle of 45° with respect to the *x*-axis shown in Fig. 1b. This excitation arrangement permits half of the excitation electric field to propagate having a polarization along the *x*-axis, with the other half of the excitation electric field propagating having a polarization along the *z*-axis. The ZnGeP_2_ crystal is excited at normal incidence, such that the excitation electric field pulse propagates along the [110] crystal direction. To permit lock-in detection, the excitation beam is mechanically chopped at a frequency of 10 kHz, prior to the beam being focussed by a 5 cm-focal length lens onto the ZnGeP_2_ crystal. Negatively chirped mirrors are used to realize an excitation pulse having a duration of 12 fs at the ZnGeP_2_ crystal. The intra-pulse DFG electric field produced in the ZnGeP_2_ crystal is collimated using a 2’’ focal length off-axis parabolic mirror and subsequently focussed onto a 125 µm-thick, (001) GaSe crystal via another 2’’ focal length off-axis parabolic mirror. The nonlinear process of electro-optic sampling within the GaSe is used to record the THz electric field waveform. The focussing parabolic mirror has a 2 mm-diameter hole to permit an 800 nm-central wavelength electric field pulse (produced via the Ti:sapphire laser) to be focussed onto the GaSe crystal by a 15 cm focal-length lens. The GaSe crystal is tilted by an angle of 65°, such that the intra-pulse DFG THz electric field experiences a refractive index influenced by both the ordinary and extraordinary GaSe refractive indices, while the probe pulse polarization experience the GaSe ordinary polarization^24^. Implementing several negatively chirped mirrors allows for a 12 fs probe pulse duration at the GaSe crystal. A retro-reflector mounted to a delay line alters the path length traveled by the probe pulse, thereby allowing adjustment to the path length difference between the probe pulse and the DFG THz electric field. The electro-optic signal is acquired via the combination of a quarter waveplate, Wollaston prism, balanced photodetector, and lock-in detection.

**Figure 4:**
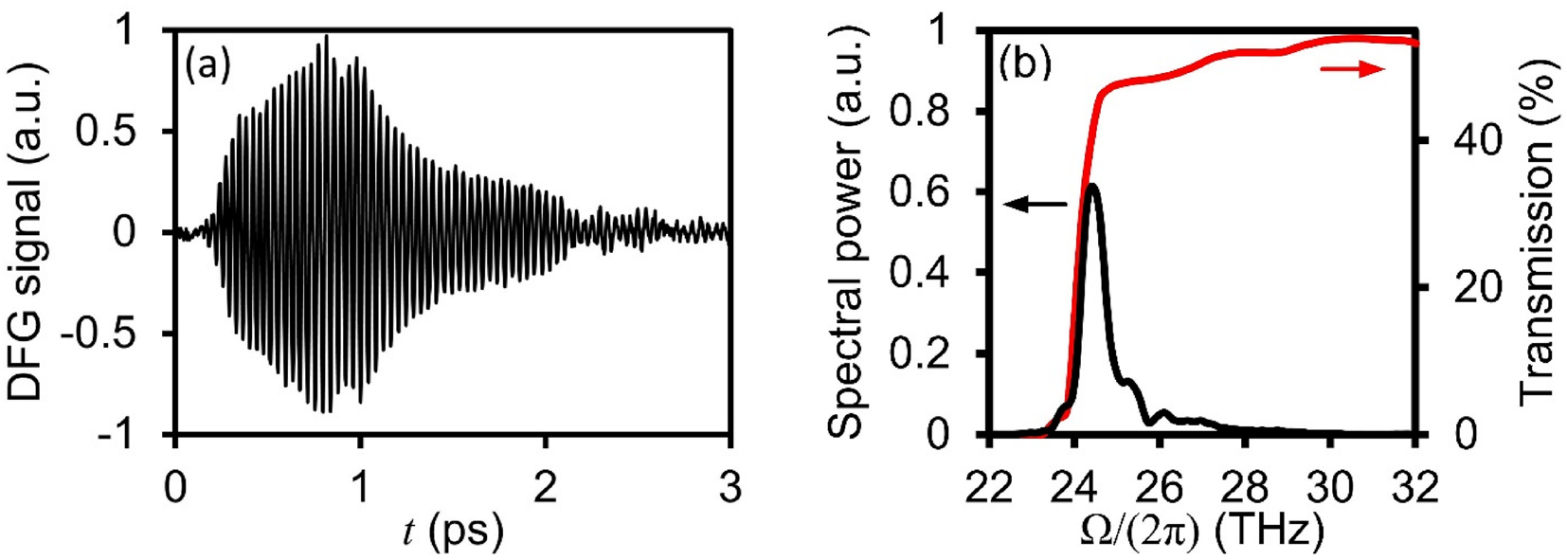
(**a**) Time-domain DFG signal due to intra-pulse mixing between the spectral components present in the near-infrared excitation pulse. (**b**) The spectral power of the intra-pulse DFG signal, shown in comparison to transmission through the ZnGeP_2_ crystal (transmission obtained from Ref. ^32^).

The time-domain intra-pulse DFG signal resulting from intra-pulse mixing is shown in Fig. 4a, which exhibits rapid oscillations (i.e., a period of ~ 40 fs) lasting several picoseconds. Interestingly, at a wavelength of 800 nm, the ordinary and extraordinary group refractive indices of the ZnGeP_2_ crystal are ${n}_{o}^{g}$=3.86 and ${n}_{e}^{g}$=3.97, respectively, such that the excitation electric field pulse polarizations temporally separate as they propagate through the crystal. To determine the propagation distance, ${L}_{s}$, at which the excitation electric field pulse polarizations have temporally separated by a duration of $\Delta t$, the following equation is used:

10 $${L}_{s}=\frac{c\Delta t}{\left|{n}_{o}^{g}-{n}_{e}^{g}\right|} .$$

When implementing the excitation pulse duration (i.e., $\Delta t$=12 fs) and twice the excitation pulse duration (i.e., $\Delta t$=24 fs), ${L}_{s}$ is calculated as 32.9 and 65.8 µm, respectively. As such, we predict that intra-pulse DFG ceases after the excitation electric field pulse propagate 32.9–65.8 µm within the ZnGeP_2_ crystal. Notably, linear optical absorption influences the excitation pulse as it propagates these distances within the ZnGeP_2_ crystal. At 800 nm, the absorption coefficient of ZnGeP_2_ along both the ordinary and extraordinary crystal axes is ~ 10 cm^−1^^30^, such that absorption of the excitation electric field pulse is only 2% and 3% after propagating the distances of 32.9 µm and 65.8 µm, respectively. The spectral power shown in Fig. 4b indicates maximum intra-pulse DFG occurs at the frequency of 24.5 THz, in excellent agreement with the calculated ${L}_{c}$ (see Fig. 2). Nonetheless, the intra-pulse DFG bandwidth spans from 23 to 30 THz with a 30 dB dynamic range, where broadband generation is due to the fact that ${L}_{c}$ > ${L}_{s}$ within this spectral range. The lower THz radiation generation limit of $\Omega /\left(2\pi \right)$= 23 THz is due to single-phonon and two-phonon absorption processes in the ZnGeP_2_ crystal^31^. As seen from Fig. 4b, the low-frequency limit agrees well with ZnGeP_2_ transmission measurements^32^. The upper THz radiation generation limit of $\Omega /\left(2\pi \right)$ = 30 is due to the fact that: (i) ${L}_{c}$<${L}_{s}$ and (ii) detection using the 65°-tilted GaSe crystal diminishes at frequencies approaching 30 THz.

It is worth considering the conversion efficiency. Intra-pulse DFG from the ZnGeP_2_ crystal is compared to that from a (001) GaSe crystal having a thickness of 250 µm, tilted by 65°, and excited using a polarization oriented 45° relative to the [001] axis. Notably, generation is only expected within the first ~ 90 µm of the crystal due to the excitation pulse polarization components experiencing different refractive indices^6^. Using the recorded electro-optic sampling signals, the intra-pulse DFG conversion efficiency from ZnGeP_2_ is calculated to be nearly an order of magnitude lower than that from GaSe. This conversion efficiency difference mainly arises from: (i) linear optical absorption in ZnGeP_2_ occurring at frequencies $\lesssim$ 23 THz and (ii) the intra-pulse DFG phase-matching condition not being satisfied in ZnGeP_2_ at frequencies $\gtrsim$ 30 THz. It would be interesting to consider excitation of the ZnGeP_2_ crystal using higher central wavelength pulses (i.e. > 800 nm) to determine if intra-pulse DFG phase-matching is possible at frequencies in the 30–40 THz range. This could improve the conversion efficiency by shifting generation further from the spectral region exhibiting linear optical absorption (i.e. $\lesssim$ 23 THz).

### Time–frequency response

**Figure 5:**
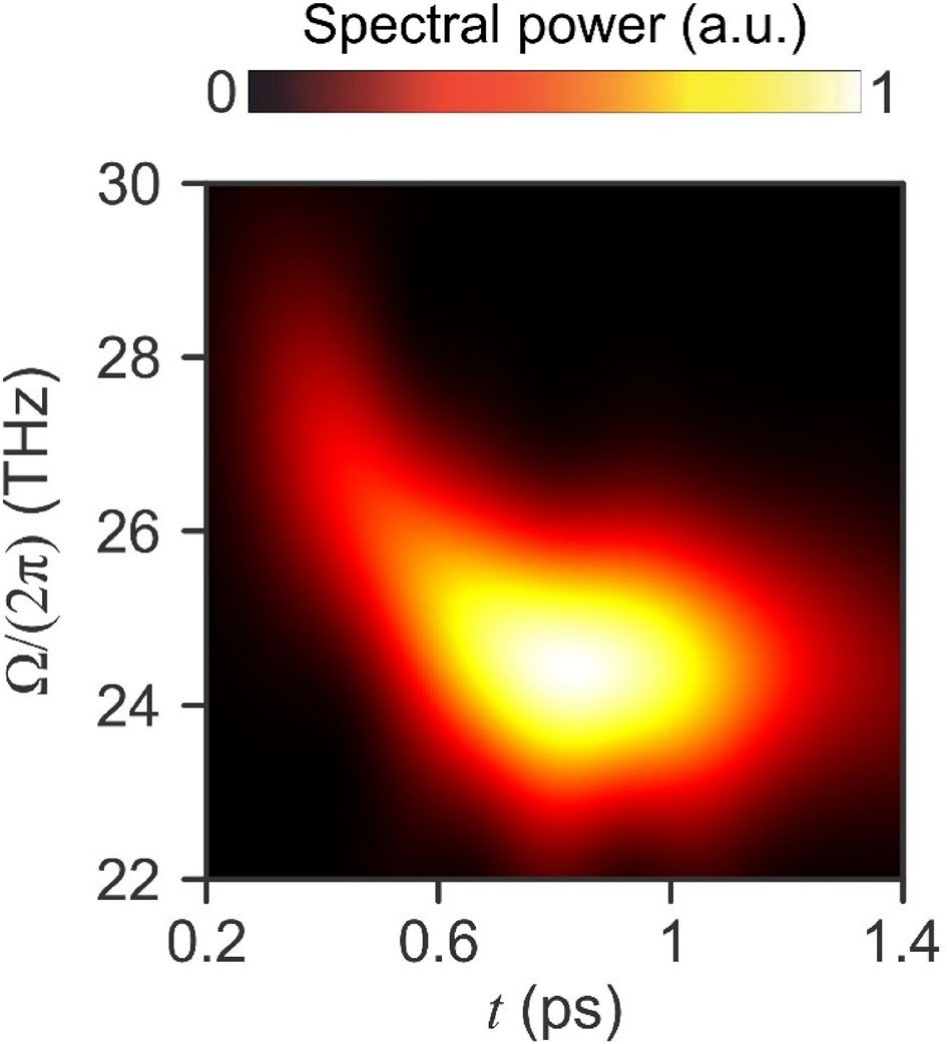
Time–frequency spectrum for the intra-pulse DFG signal produced by the ZnGeP_2_ crystal.

Since the intra-pulse DFG signal has a long duration of several picoseconds, exhibits ~ 50 periods within its envelope, and encompasses a wide range of spectral components, it is instructive to consider the time–frequency spectrum of the intra-pulse DFG signal (see Fig. 5). Interestingly, the higher-frequency THz electric field components [e.g., $\Omega /\left(2\pi \right)$≈ 28 THz] arrive several hundreds of femtoseconds prior to the lower-frequency THz electric field components [e.g., $\Omega /\left(2\pi \right)$ ≈ 24 THz]. This is due to the electric field components at $\Omega /\left(2\pi \right)$ ≈ 28 THz experiencing a lower ${n}_{o}^{g}$ in comparison to the $\Omega /\left(2\pi \right)$≈ 24 THz electric field components. Specifically, after intra-pulse DFG occurs in the initial ${L}_{s}$=32.9–65.8 µm of the crystal, the $\Omega /\left(2\pi \right)$ ≈ 28 THz electric field components propagate through the remaining > 900 µm of the crystal at a higher group velocity (i.e., lower ${n}_{o}^{g}$) than the $\Omega /\left(2\pi \right)$≈ 24 THz electric field components. Given this, along with intra-pulse DFG only occurring within the initial 32.9–65.8 µm of the crystal, a thinner ZnGeP_2_ crystal would provide a shorter-duration THz electric field pulse with a similar or higher energy. While strong oscillations at $\Omega /\left(2\pi \right)$ ≈ 24 THz exist between *t* = 0.6–1.3 ps (indicated by a strong spectral power in Fig. 5), lower-strength oscillation at this frequency persist until *t *≈ 2.1 ps. The existence of oscillations having a duration of several picoseconds indicates that intra-pulse DFG is occurring near a resonance (e.g., single-phonon or multi-phonon) frequency supported by the crystal^33,34^. In such a scenario, the excitation laser pulse induces nonlinear dipole oscillations near the resonance frequency of the crystal, with the induced dipoles exhibiting a damping time that is longer than the duration of the excitation pulse^33,34^. The several-picosecond oscillations observed at $\Omega /\left(2\pi \right)$≈ 24 THz occur near a two-phonon process in the ZnGeP_2_ crystal^31^, such that this two-phonon resonant process is attributed to the long-duration THz electric field generation.

## Conclusion

A chalcopyrite ZnGeP_2_ crystal cut along the (110) crystallographic plane is used to realize a phase-resolved electric field pulse encompassing frequencies between 23-30 THz and exhibiting a maximum spectral power at 24.5 THz. The maximum generation frequency agrees with intra-pulse DFG phase-matching calculations, with phase-matching being achieved using an excitation electric field pulse having polarization components along both the ordinary and extraordinary crystal axes. It would be interesting to consider ZnGeP_2_ for the electro-optic detection of frequencies within this same frequency range of 23–30 THz, potentially allowing for a high-frequency THz system that uses ZnGeP_2_ as both the intra-pulse DFG emitter crystal and the electro-optic sensor crystal.

## Data Availability

The data that support the findings of this study are available from the corresponding author upon reasonable request.
